# Scorpion Neurotoxin Syb-prII-1 Exerts Analgesic Effect through Nav1.8 Channel and MAPKs Pathway

**DOI:** 10.3390/ijms23137065

**Published:** 2022-06-25

**Authors:** Fei Bai, Yongbo Song, Yi Cao, Mengqi Ban, Zhenyu Zhang, Yang Sun, Yuan Feng, Chunli Li

**Affiliations:** 1 Department of Pharmacology, Shenyang Pharmaceutical University, Shenyang 110016, China; 15653250971@163.com (F.B.); caoyi940216@163.com (Y.C.); banmonkey1@126.com (M.B.); 18609831621@163.com (Y.S.); fengyuan_22@outlook.com (Y.F.); 2 Department of Biochemistry, Shenyang Pharmaceutical University, Shenyang 110016, China; songyongbo@syphu.edu.cn (Y.S.); v18306510590@163.com (Z.Z.)

**Keywords:** trigeminal neuralgia, Syb-prII-1, β-type scorpion neurotoxin, BmK, VGSCs, Nav1.8, Nav1.9, IoN-CCI model, MAPKs pathway, VSD2^rNav1.8^

## Abstract

Trigeminal neuralgia (TN) is a common type of peripheral neuralgia in clinical practice, which is usually difficult to cure. Common analgesic drugs are difficult for achieving the desired analgesic effect. Syb-prII-1 is a β-type scorpion neurotoxin isolated from the scorpion venom of *Buthus martensi Karsch (BmK).* It has an important influence on the voltage-gated sodium channel (VGSCs), especially closely related to Nav1.8 and Nav1.9. To explore whether Syb-prII-1 has a good analgesic effect on TN, we established the Sprague Dawley (SD) rats’ chronic constriction injury of the infraorbital nerve (IoN-CCI) model. Behavioral, electrophysiological, Western blot, and other methods were used to verify the model. It was found that Syb-prII-1 could significantly relieve the pain behavior of IoN-CCI rats. After Syb-prII-1 was given, the phosphorylation level of the mitogen-activated protein kinases (MAPKs) pathway showed a dose-dependent decrease after IoN-CCI injury. Moreover, Syb-prII-1(4.0 mg/kg) could significantly change the steady-state activation and inactivation curves of Nav1.8. The steady-state activation and inactivation curves of Nav1.9 were similar to those of Nav1.8, but there was no significant difference. It was speculated that it might play an auxiliary role. The binding mode, critical residues, and specific interaction type of Syb-prII-1 and VSD2^rNav1.8^ were clarified with computational simulation methods. Our results indicated that Syb-prII-1 could provide a potential treatment for TN by acting on the Nav1.8 target.

## 1. Introduction

TN is typically a chronic neuralgia that can be triggered by touching the face [1,2,3]. TN mostly occurs in people over 50 years old, and the incidence rate in women is higher than that in men. There are two hypotheses about the pathogenesis of trigeminal neuralgia: central pathogenesis and peripheral pathogenesis. The viewpoint of central pathogenesis theory is the epilepsy theory and central sensitization. Peripheral disease theory can be divided into the vascular compression theory and demyelination theory. The demyelination theory is a more recognized direction. It believes that the trigeminal afferent neurons have pathological changes, the neuronal cell bodies are overexcited, and abnormal discharge occurs. However, due to the loss of myelin sheath, the nerve fibers are closely attached, that is, non-nociceptive nerve fibers Aβ and nociceptive nerve fibers Aδ, C are transmitted directly, and the pain signal is amplified in the transmission process. Therefore, slight pain stimulation in clinical practice can cause great pain. There is currently no ideal treatment in clinical practice [4,5,6]. The IoN-CCI model of TN is the first and most common model created by Vos et al. [7]. Since then, the model has been continuously improved [8]. Currently, trigeminal ganglion (TG) infraorbital nerve branches can be exposed by three surgical methods: intraoral, extraoral, paranasal [9] and brow arch [10]. We ligated the infraorbital nerve with medical chromium catguts to produce a vascular compression effect on the nerve. It may cause degeneration and necrosis of the trigeminal nerve and demyelinating lesions, leading to symptoms of pain in the mouth and face of rats.

The underlying mechanism of TN is still unclear. Animal model studies have found that the affected trigeminal nerve has channelopathies and electrophysiological abnormalities. Electrophysiological records provide new clues for the treatment of TN. VGSCs play an important role in the process of neuropathic pain [11]. It has been found that Nav1.3, Nav1.7, Nav1.8, and Nav1.9 are closely related to neuropathic pain [12]. Nav1.3 is involved in the formation of tactile allodynia and hyperalgesia [13]. Nav1.8 and Nav1.9 are specifically expressed on pain-sensing neurons of TG and dorsal root ganglia (DRG) and play a key role in peripheral neuropathic pain [14,15,16]. Nav1.8 is involved in the generation and transduction of pain signals, and Nav1.8 specific channel blockers can provide analgesic effects in inflammatory pain models and neuropathic pain models [17,18]. Nav1.8 can participate in the process of up- and down-regulation of pain [19]. Nav1.8 is mainly involved in the formation of the rising branch of the action potential of neuron nociceptors, and it can regulate the excitability of the receptor in a low temperature environment. Nav1.9 can regulate resting membrane potential and prolong the depolarization response of subthreshold stimulation to reduce the threshold and increase repetitive stimulation [20,21]. In addition, studies have shown that many bio-peptide drugs exert analgesic effects by regulating the function of various ion channels. Therefore, various subtypes of VGSCs can serve as molecular targets for the development of novel analgesic drugs [22].

Syb-prII-1 is a β-type scorpion neurotoxin isolated from the scorpion venom of *BmK*. It has an important influence on the VGSCs, especially ones closely related to Nav1.8 and Nav1.9. Our previous study found that Syb-prⅡ-1 could block the current of the Nav1.8 channel, and the IC_50_ of Syb-prⅡ-1 was 133.42 nM [23].

## 2. Results

### 2.1. Behavioral and Histopathological Changes

#### 2.1.1. Accelerated Rotarod and Gait Analysis

**Figure 1:**
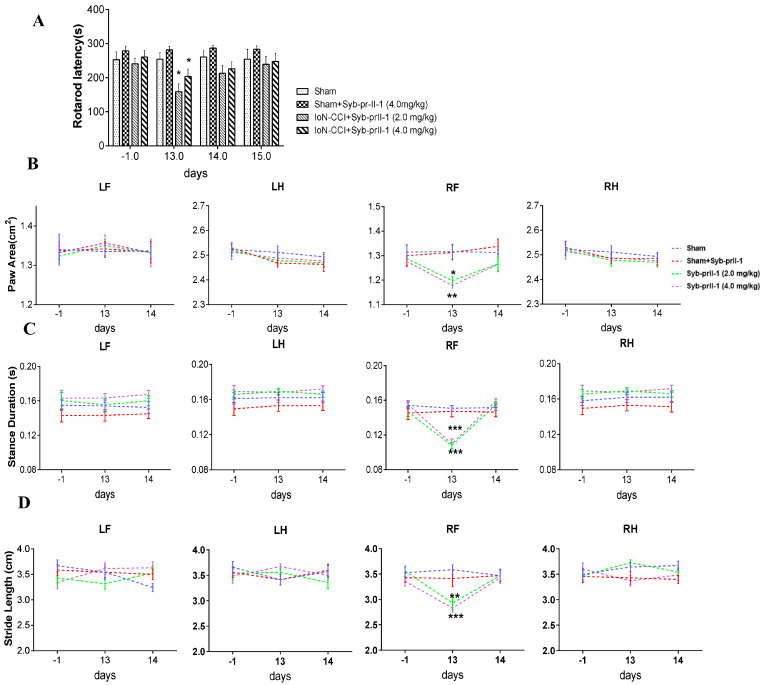
Changes in accelerated rotarod and gait analysis. (**A**) The effect of Syb-prII-1 on the accelerated rotarod experiment of IoN-CCI rats (*n* = 12). (**B**) Effects of Syb-prII-1 on paw area following IoN-CCI operation in rats (*n* = 8). (**C**) Effects of Syb-prII-1 on stance duration following IoN-CCI model established in rats (*n* = 8). (**D**) Effects of Syb-prII-1 on stride length following IoN-CCI model established in rats (*n* = 8). All data were presented as mean ± SEM. * *p* < 0.05, ** *p* < 0.01, *** *p* < 0.001 compared with sham group.

Since Syb-prII-1 is a recombinant polypeptide of scorpion neurotoxin, to verify whether high-dose Syb-prII-1 can cause ataxia in animal motor function and affect subsequent pharmacodynamics and behavioral experiments, the gait analysis system was used to analyze the effects of Syb-prII-1 on experimental animals. The results showed that Syb-prII-1 did not affect the rats’ motor coordination ability (Figure 1A). At 13 days after IoN-CCI, the maximum paw area on the front paw (RF) of the model group decreased compared to the sham group, and it decreased in all treatment groups. After administration, the largest paw area of the model group returned to the preoperative detection level (Figure 1B). At 13 days after modeling, the stance duration of the ipsilateral side (RF) of the model group was significantly lower than the sham group. After administration, the ipsilateral side (RF) of the model group returned to the preoperative level and was comparable to the sham group (Figure 1C). At 13 days after the model was established, the distance between the consecutive steps of the ipsilateral front paw (RF) became smaller, but after administration, the stride length of the ipsilateral front paw (RF) returned to a level comparable to the sham group (Figure 1D).

#### 2.1.2. Mechanical Allodynia and Thermal Pain Results

**Figure 2:**
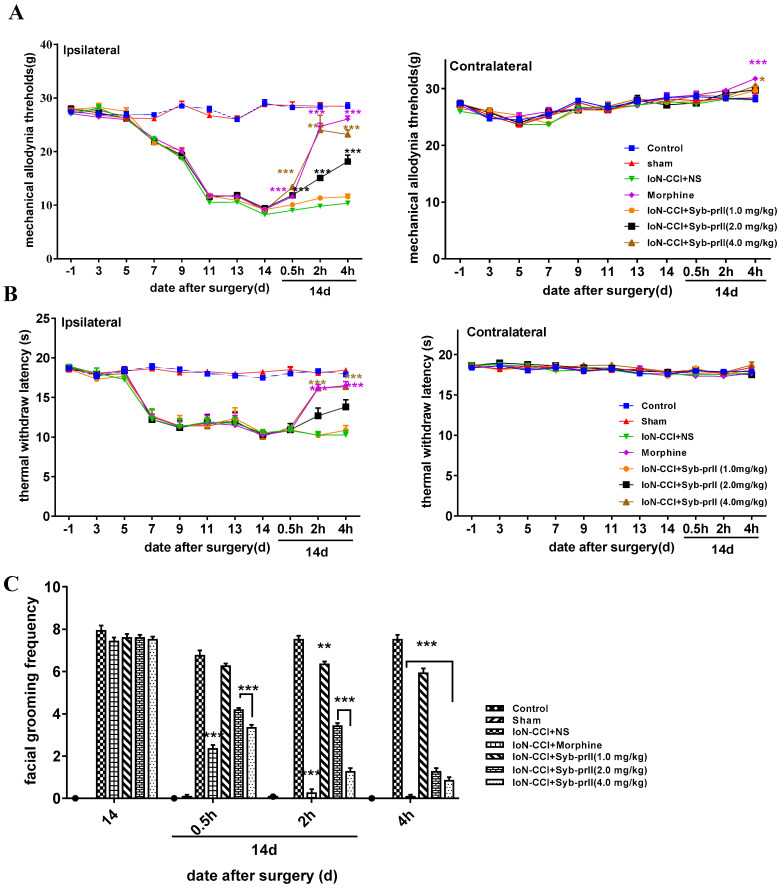

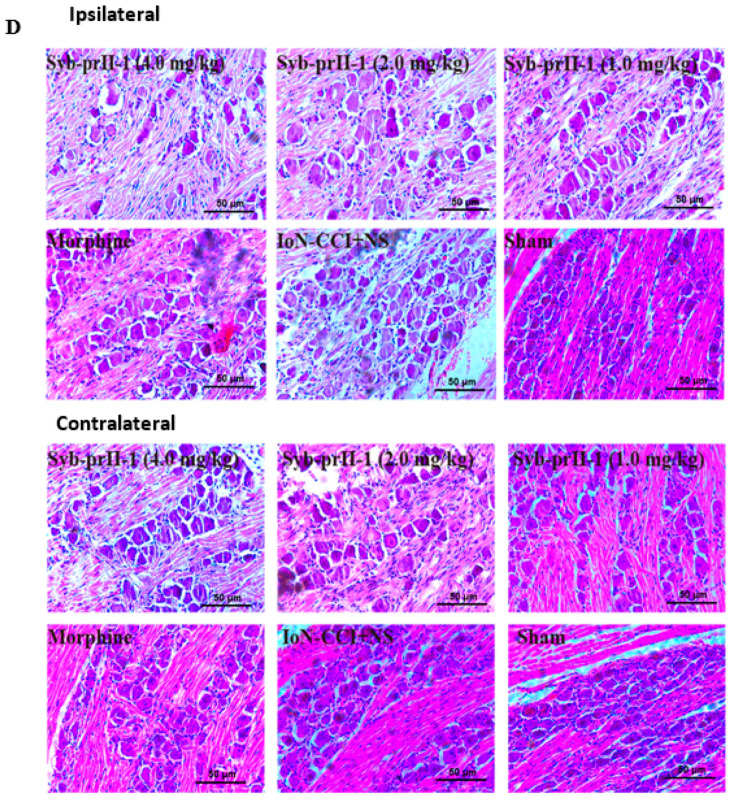
Behavioral changes in rats after IoN-CCI model. (**A**) Changes of mechanical allodynia thresholds in response to the mechanical stimuli in the area of rats’ whisker pads. (**B**) Changes of thermal withdrawal latencies in the area of rats’ right whisker pads after gradient doses of Syb-prII-1 treatment in rats. (**C**) Changes of scores of spontaneous asymmetrical facial grooming on the rats’ right whisker pads caused by Syb-prII-1 and morphine. All data were presented as mean ± SEM. *n* = 12. * *p* < 0.05, ** *p* < 0.01, *** *p* < 0.001 compared with model group. (**D**) Histological examination of trigeminal neural ganglion from ipsilateral and contralateral sides in every group (×400, HE staining).

After modeling, the mechanical allodynia threshold of the rats decreased significantly and reached the lowest level on the 14th day, after Syb-prII-1 administration at 14 days. The analgesic effect of high dose was the most significant, and the maximum analgesic effect appeared at around 2 h. It had an analgesic effect comparable to morphine (Figure 2A). The thermal withdrawal latency reached the shortest level on the 14th day. After Syb-prII-1 administration, the high-dose group exerted an analgesic effect (Figure 2B). The facial grooming frequency of rats was shown in Figure 2C. After 0.5 h, the inhibition rates of Syb-prⅡ-1 at 1.0 mg/kg, 2.0 mg/kg, and 4.0 mg/kg were 7.41%, 38.22%, and 50.67%; after 2 h, the inhibition rates were 15.60%, 54.40%, and 83.33%; The inhibition rates after 4 h were 21.07%, 83.33%, and 88.93%.

#### 2.1.3. Pathological Changes of the Trigeminal Nerve

HE staining of the trigeminal nerve found that the sham group on the ipsilateral side had complete cell membranes, clear nuclei, and uniform chromatin distribution. Most neurons in the model group experienced cell shrinkage, void necrosis, and uneven distribution of chromatin. The morphine group and different dose of Syb-prII-1 groups could not improve this state. The morphology of neurons in the contralateral group was normal, without significant changes (Figure 2D).

### 2.2. The Expression of Nav1.8 and Nav1.9 in TG Neurons

**Figure 3:**
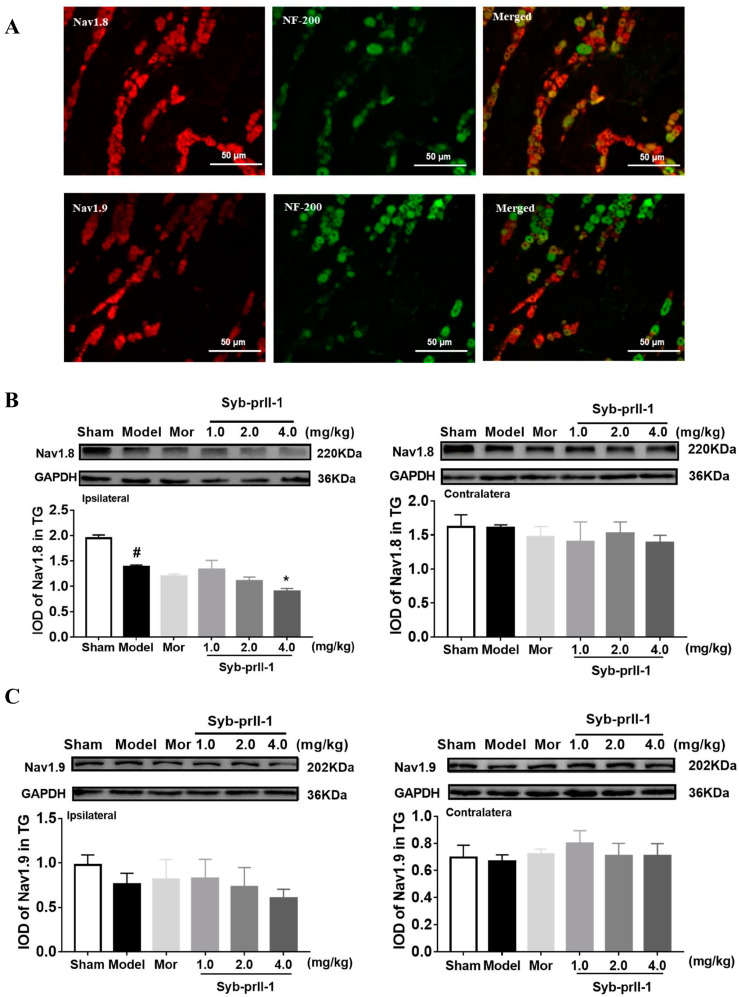
Expression of Nav1.8 and Nav1.9 in TG neurons. (**A**) TG neurons were labeled with anti-NF200 and anti-Nav1.8/anti-Nav1.9 antibodies. The panel in the columns marked “Merged” is the merged image of the panel in the two left columns. (**B**) Effects of Syb-prII-1 on the expression of Nav1.8 in different groups of trigeminal ganglia. There was a significant decrease in the expression level of Nav1.8 after Syb-prⅡ-1(4.0 mg/kg) treatment. (**C**) Effects of Syb-prII-1 on the expression of Nav1.9 in different groups of trigeminal ganglia. There was no significant decrease in the expression level of Nav1.9 after Syb-prⅡ-1 treatment. All data were presented as mean ± SEM. *n* = 3. * *p* < 0.05 compared with the model group. ^#^ *p* < 0.05 compared with sham group.

The mouse anti-NF200 monoclonal antibody was incubated with rabbit anti-Nav1.8 and Nav1.9 monoclonal antibodies, respectively, to determine the distribution of Nav1.8 and Nav1.9 ion channels. Figure 3A shows that the markers Nav1.8 (red) and NF200 (green) only slightly overlap, indicating that Nav1.8 and Nav1.9 are mainly distributed on small and medium-diameter neurons, and rarely on large-diameter neurons. After the IoN-CCI model was established, the expression of Nav1.8 and Nav1.9 in rat TG neurons was observed. The expression of Nav1.8 protein in the model group was significantly reduced. There was no significant effect on the morphine group. The expression of Nav1.8 protein was significantly decreased in the 4.0 mg/kg Syb-prII-1 group (Figure 3B). The expression of Nav1.9 protein in each group did not change significantly (Figure 3C).

### 2.3. The Changes of MAPKs Pathway

**Figure 4:**
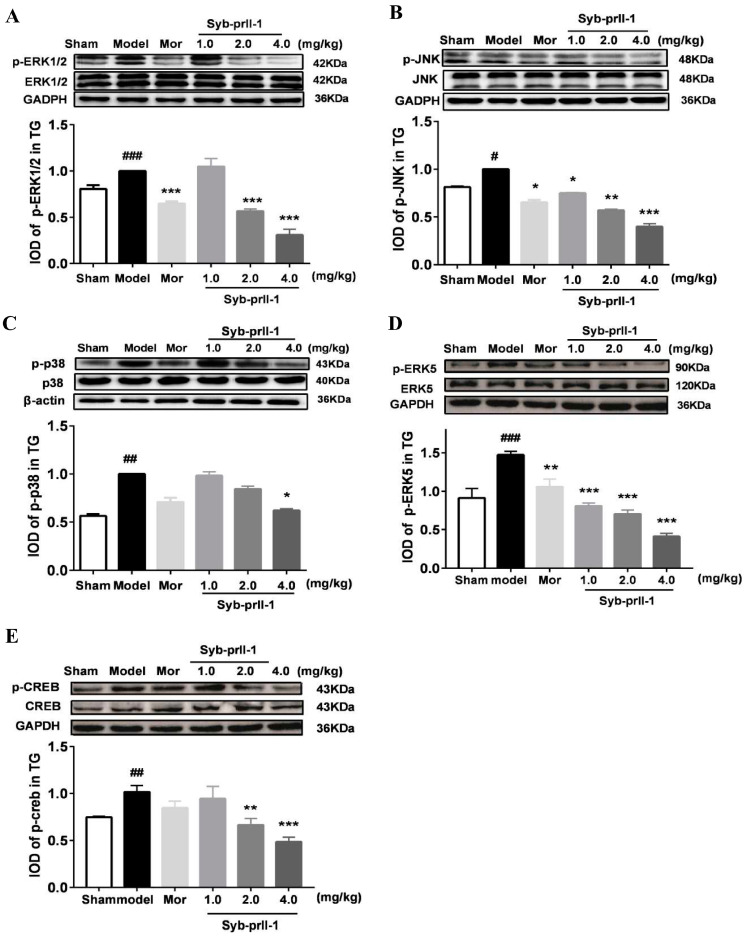
Phosphorylation levels of MAPKs pathway proteins. (**A**) Effects of Syb-prII-1 on phosphorylation of ERK1/2 in different groups of trigeminal ganglia. (**B**) Effects of Syb-prII-1 on phosphorylation of JNK in different groups of trigeminal ganglia. (**C**) Effects of Syb-prII-1 on phosphorylation of P38 in different groups of trigeminal ganglia. (**D**) Effects of Syb-prII-1 on phosphorylation of ERK5 in different groups of trigeminal ganglia. (**E**) Effects of Syb-prII-1 on phosphorylation of CREB in different groups of trigeminal ganglia. All data were presented as mean ± SEM. *n* = 3. * *p* < 0.05, ** *p* < 0.01, *** *p* < 0.001 compared with the model group. ^#^ *p* < 0.05, ^##^ *p* < 0.01, ^###^ *p* < 0.001 compared with sham group.

After different doses of Syb-prII-1 were administered, the phosphorylation levels of the four pathways of c-Jun N-terminal kinase (JNK), extracellular regulated protein kinases1/2 (ERK1/2), p38, and extracellular-regulated kinase 5 (ERK5) decreased in a dose-dependent manner (Figure 4). The CREB phosphorylation level of the model group was significantly higher than that of the sham group. Syb-prII-1 significantly inhibited the phosphorylation of CREB in a dose-dependent manner.

### 2.4. Electrophysiological Results

#### 2.4.1. Effect of Syb-prII-1 on Nav1.8 and Nav1.9 Current Amplitude of TG Neurons

**Figure 5:**
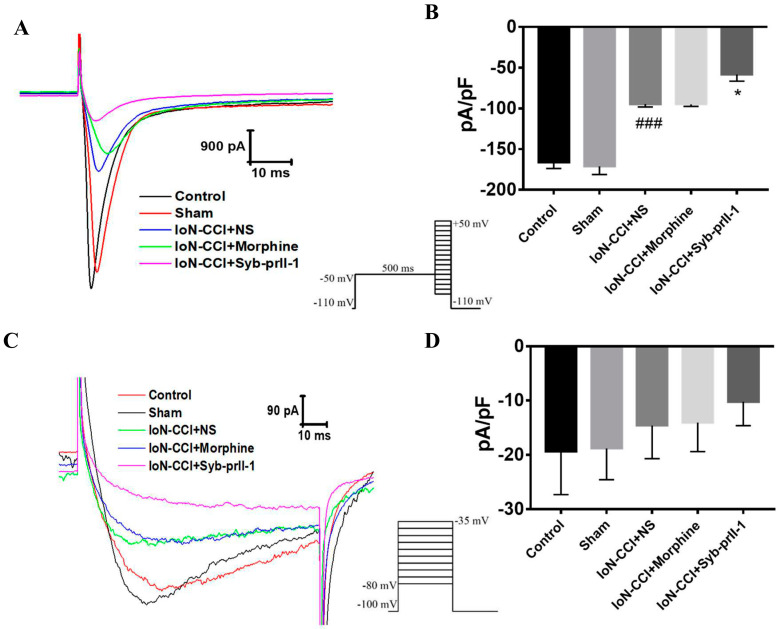
Changes in peak currents of Nav1.8 and Nav1.9 after IoN-CCI model and Syb-prII-1 injection. (**A**) The Nav1.8 current was evoked by a depolarizing voltage step from −50 mV to +50 mV in 10 mV increments from a holding potential of −100 mV (*n* = 9). (**B**) Effect of Syb-prII-1 on Nav1.8 current density in TG neurons (*n* = 9). (**C**) The Nav1.9 current was evoked by a single voltage step at −35 mV from a holding potential of −100 mV (*n* = 9). (**D**) Effect of Syb-prII-1 on Nav1.9 current density in TG neurons. Data were shown as mean ± SEM. ^###^ *p* < 0.001 compared with sham group. * *p* < 0.05 compared with model group.

Statistical analysis was performed on the Nav1.8 peak current on the ipsilateral side of the rats. Compared with the control group, the current density of the model group was significantly reduced. Compared with the model group, the Nav1.8 peak current density in the morphine group did not change significantly. The peak current density of Nav1.8 was significantly reduced after Syb-prII-1 (4.0 mg/kg) was administered (Figure 5A,B). 

Statistical analysis was performed on the Nav1.9 peak current on the ipsilateral side of the rats. Compared with the control group, the current density of the model group decreased slightly, but there was no statistical difference. There was no significant effect on Nav1.9 current density after morphine administration. After Syb-prII-1 (4.0 mg/kg) was given, the current of Nav1.9 was inhibited to some extent, but there was no significant difference (Figure 5C,D). 

#### 2.4.2. I-V Curve and Steady-State Activation Curve of Nav1.8 and Nav1.9 Currents

**Figure 6:**
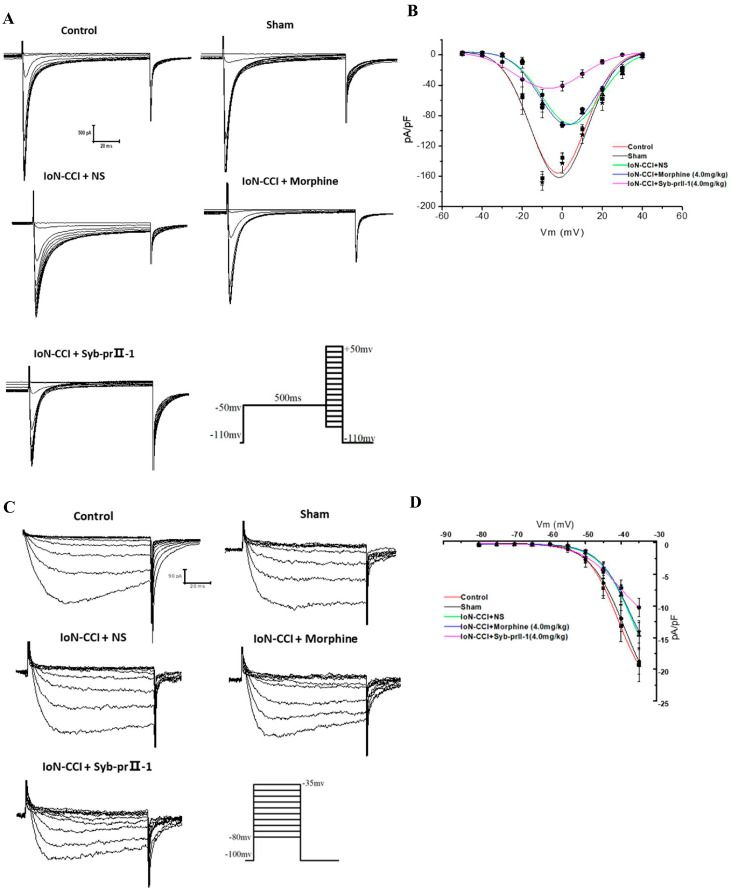

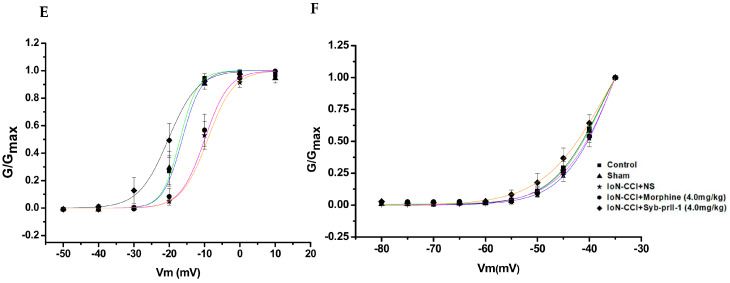
Changes in Nav1.8 and Nav1.9 current I-V curves and steady-state activation curves in TG neurons after IoN-CCI treatment and Syb-prⅡ-1 injection. (**A**) A series of Nav1.8 currents recorded in the presence of 1000 nM TTX. Cells were depolarized to a variety of potentials (−50 mV to +50 mV) from a holding potential of −100 mV to elicit Nav1.8 currents (*n* = 9). (**B**) I-V curves of Nav1.8 currents. (**C**) A series of Nav1.9 currents recorded in the presence of 1000 nM TTX. Cells were depolarized to a variety of potentials (−80 mV to −35 mV) from a holding potential of −100 mV to elicit Nav1.9 currents (*n* = 9). (**D**) I-V curves of Nav1.9 currents. (**E**) Changes in the steady-state activation curves of Nav1.8 in TG neurons. IoN-CCI surgery shifted the steady-state activation curve in a depolarization direction; 4.0 mg/kg Syb-prⅡ-1 shifted the steady-state activation curve in a hyperpolarizing direction (*n* = 9). (**F**) Changes in the steady-state activation curves of Nav1.9 in TG neurons IoN-CCI surgery shifted the steady-state activation curve in a depolarization direction; 4.0 mg/kg Syb-prⅡ-1 shifted the steady-state activation curve in a hyperpolarizing direction (*n* = 9).

A series of Nav1.8 currents were recorded according to the protocol. The peak current density measured under the stimulation pulse under different conditions was the ordinate, and the corresponding conditional stimulation pulse voltage was the abscissa. Plot the Nav1.8 current I-V curve (Figure 6A,B). On the ipsilateral side, the I-V curve of the model group was significantly up-shifted compared to the control group. The morphine group had no significant effect on the I-V curve compared to the model group. When given the Syb-prII-1 (4.0 mg/kg), the Nav1.8 current I-V curve continued to shift upward, and the peak current activation voltage dropped back to −10 mV; the reversal potential of the Syb-prII-1 group changed significantly. In the Nav1.9 current I-V curve, the model group I-V curve was slightly up-shifted compared to the control group. The Nav1.9 current I-V curve continued to show a slight up-shift after administration of Syb-prII-1 (4.0 mg/kg) compared to the model group. There were no significant changes in the other groups (Figure 6C,D).

The Boltzmann equation $G∕=1∕\left\{1+exp[\left(V_{1∕2\mathrm{act}}-V\right)∕k]\right\}$ was used to obtain the Nav1.8 steady-state activation curve. Compared with the control group, the model group Nav1.8 current steady-state activation curve drifted significantly in the direction of depolarization. Compared with the model group, morphine had no significant effect. After Syb-prⅡ-1 (4.0 mg/kg) was administered, the Nav1.8 current steady-state activation curve drifted significantly toward the hyperpolarization direction. The half activation voltage *V*_1/2act_ and slope factor *k* of each group were −17.41 ± 0.12, 2.60 ± 0.10; −17.17 ± 0.34, 3.20 ± 0.27; −10.26 ± 0.24, 3.79 ± 0.27; −11.00 ± 0.13, 3.74 ± 0.14; −20.16 ± 0.31, 4.64 ± 0.30 (Figure 6E). Fitting the Boltzmann equation can obtain the Nav1.9 steady-state activation curve. The results showed that the Nav1.9 steady-state activation curves of the control group, sham group, model group, and morphine group did not change significantly. After Syb-prⅡ-1 was given, the Nav1.9 activation curve shifted slightly toward the direction of hyperpolarization. The *V*_1/2act_ and *k* of each group were −37.65 ± 0.77, 4.85 ± 0.25; −36.89 ± 0.79, 4.97 ± 0.24; −34.86 ± 1.45, 4.90 ± 0.34; −33.41 ± 2.96, 5.50 ± 0.59; −36.76 ± 1.33, 6.10 ± 0.37 (Figure 6F).

#### 2.4.3. Steady-State Inactivation Curves of Nav1.8 and Nav1.9

**Figure 7:**
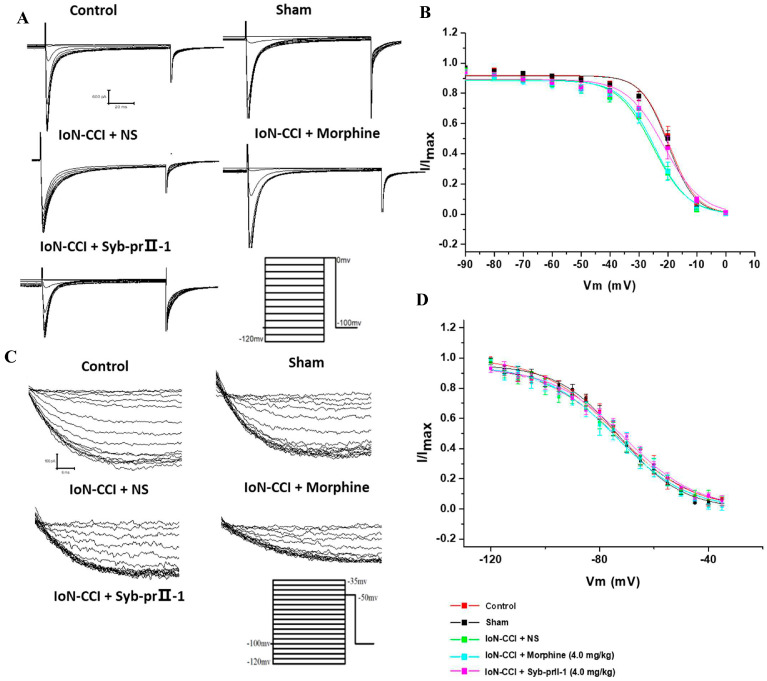
Effects of Syb-prII-1 on steady-state inactivation curves of Nav1.8 and Nav1.9. (**A**) Nav1.8 inactivation current was generated in 10 mV progressive steps between −120 mV and 0 mV from a holding potential of −100 mV. (**B**) Nav1.8 steady-state inactivation curve. IoN-CCI surgery shifted the steady-state inactivation curve in a hyperpolarizing direction; 4.0 mg/kg Syb-prⅡ-1 shifted the steady-state inactivation curve in a depolarization direction (*n* = 9). (**C**) The protocol to elicit Nav1.9 inactivation currents starting from a holding potential of −100 mV, applying conditioning pulses ranging from −110 mV to −35 mV in increments of 5 mV, and applying a test pulse at −50 mV. (**D**) Nav1.9 steady-state inactivation curve IoN-CCI surgery shifted the steady-state inactivation curve in a hyperpolarizing direction; 4.0 mg/kg Syb-prⅡ-1 shifted the steady-state inactivation curve in a depolarization direction (*n* = 6).

The Boltzmann equation $I∕=1∕\left\{1+exp\left[\left(V-V_{1∕2\;\mathrm{inact}}\right)∕k\right]\right\}$ was used to fit the Nav1.8 steady-state inactivation curve. It could be seen from the results that compared with the control group, the Nav1.8 current inactivation curve of TG neurons in the sham group did not change significantly, and the inactivation curve of the model group drifted toward the hyperpolarization direction; after Syb-prⅡ-1 (4.0 mg/kg) was given, the Nav1.8 current inactivation curve drifted toward the depolarization direction. The half inactivation voltage *V*_1/2 inact_ and *k* of each group were −20.02 ± 0.88, 5.23 ± 0.80; −20.38 ± 0.90, 5.11 ± 0.82; −26.17 ± 1.27, 7.07 ± 1.10; −25.41 ± 1.16, 6.64 ± 1.00; −22.22 ± 1.19, 7.32 ± 1.04 (Figure 7A,B).

The Boltzmann equation was used to fit the Nav1.9 steady-state inactivation curve. The results showed that there was no significant change in the Nav1.9 inactivation curve in each group. The *V*_1/2 inact_ and *k* of each group were −73.80 ± 0.70, 13.67 ± 0.64; −74.20 ± 0.53, 12.68 ± 0.48; −75.35 ± 0.64, 15.45 ± 0.61; −75.97 ± 0.73, 14.44 ± 0.68; 73.44 ± 0.70, 15.19 ± 0.66 (Figure 7C,D). 

### 2.5. The Result of Homology Modeling and Molecular Docking of Syb-prII-1 and VSD2^rNav1.8^

Human Nav1.2-beta2-KIIIA (PDB ID: 6J8E), which has 65.96% sequence identity with rNav1.8 (Uniprot accession number Q9Y5Y9) [24], was the best template for building the VSD2^rNav1.8^ structure. The antidepressant anti-insect neurotoxin (PDB ID: 2I61) was selected as the template for Syb-prII-1 structure modeling based on 78.69% sequence identity.

**Figure 8:**
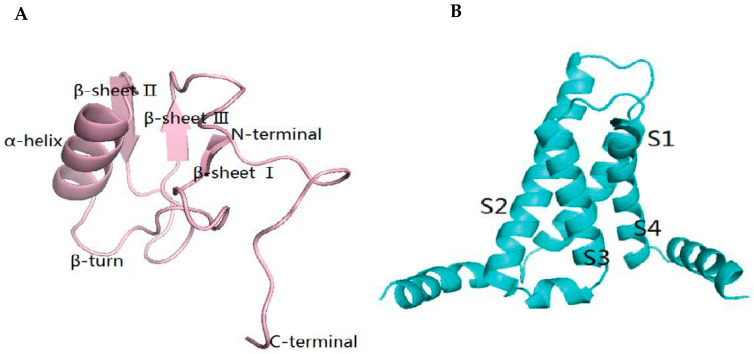
(**A**) Homology modeling of Syb-prII-1. (**B**) Homology modeling of VSD2^rNav1.8^.

The results of the ERRAT method were that the overall quality factors of Syb-prII-1 and VSD2^rNav1.8^ were 80.4878 and 96.6387, respectively. Both results were higher than the standard required by the method. The amino acid residues of Syb-prII-1 and VSD2^rNav1.8^ in the optimal conformation were 93.8% and 92.1% in the optimum region, and 6.2% and 7.9% in the allowable region. It could be seen that the proportion of amino acid residues of Syb-prII-1 and VSD2^rNav1.8^ in the maximum allowable region exceeds 90%, and the conformation of the model is considered to be in accordance with the rules of stereochemistry. Moreover, the two models passed the overall summary report of WHATCHECK. Based on the above, reasonable simulation structures of Syb-prII-1 and VSD2^rNav1.8^ were obtained (Figure 8A,B).

### 2.6. The Result of Molecular Dynamics Simulation

**Figure 9:**
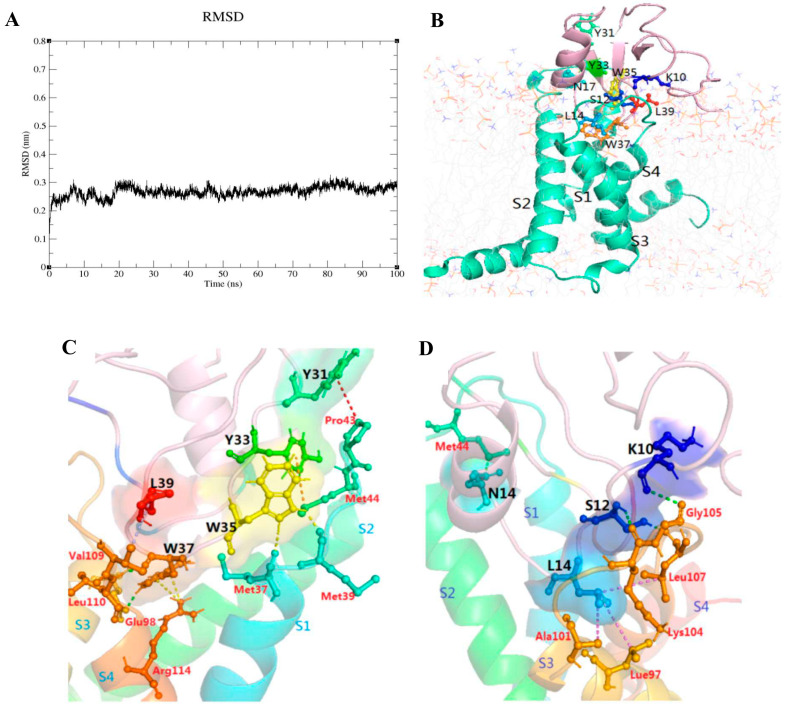
(**A**) RMSD curves of the complex systems of VSD2^rNav1.8^-Syb-prII-1. (**B**) The structure of the complexes in the membrane after MD simulation. Binding modes of related residues on IS1 (**C**) and IS2 (**D**) Names of residues on the peptide are in black; names of residues on VSD2^rNav1.8^ are in red.

The trajectories analysis of 100 ns MD simulation was applied to calculate the RMSD of backbone atoms from the complex systems of VSD2^rNav1.8^-Syb-prII-1. As shown in Figure 9A, the average RMSD value was 0.27 nm, and this system reached a stable state in 90–100 ns. The complex structure at this stage reflected the best final binding mode of VSD2^rNav1.8^ and Syb-prII-1, so we selected it to perform further analysis. The residues located on the binding surface of VSD2^rNav1.8^ with Syb-prII-1 were shown in Figure 9B. Observed from the spatial structures of VSD2^rNav1.8^-Syb-prII-1 in Figure 9B, there were nine amino acid residues of Syb-proII-1 on the binding surface of VSD2^rNav1.8^-Syb-prII-1. These residues were distributed mainly in α-helix (N17), the loop between α-helix and β-sheet I (K10, S12, L14), and β-turn (Y31, Y33, W35, W37, L39) of Syb-prII-1. 

The binding mode of the residues involved in the interaction was shown in Table 1 and Figure 9C,D. K10, S12, L14 on Syb-prII-1 were capable of contacting with the loop connecting DII/S3-S4 to VSD2^rNav1.8^ and formed the first main interaction surface (called IS1). In addition, the interactions betweenY31/Y33/W35/W37/L39 on Syb-prII-1 and Met37/Met39/Pro43/Met44/Glu98/Val109/Leu110/Arg114 on VSD2^rNav1.8^ constituted the other interaction surface (called IS2).

Regarding IS1, S12 on Syb-prII-1 had interactions with Lys104 and Leu107 on VSD2^rNav1.8^ by hydrogen bond, and L14 on Syb-prII-1 was combined with Leu97, Ala101, and Leu107 on VSD2^rNav1.8^ by hydrophobic bond. Then, we predicted that S12 and L14 played a key role in binding VSD2^rNav1.8^ with Syb-prII-1. Meanwhile, Lys104 was the main contributor to the interaction, which was consistent with the previous finding [25].

As for IS2, the conserved W35 and W37 was also an important contributor to interactions between Syb-prII-1 and VSD2^rNav1.8^. W37 on Syb-prII-1 was able to form hydrogen bonds with Leu110 and Glu98 on VSD2^rNav1.8^. W37 also formed two Pi-Cation interactions with Arg114 on VSD2^rNav1.8^, which was one of four highly conserved arginines located in S4. Four arginines on S4 made major contributions to promoting VSD trapping [26]. We speculated that W37 could play a key role in binding to VSD2^rNav1.8^ and further affecting its function. Moreover, we found that tryptophans distributed around W37 were highly conserved and contributed to the binding of β-ScTx and VGSC. Our results also found that W35 also formed two hydrogen bonds with Met39 and Met37 on VSD2^rNav1.8^. This result was consistent with our previous results, where the W38 residue of AGAP exerted a crucial influence on the interaction of *BmK* AGP-SYPU1 with human Nav1.7 [27]. Furthermore, it was easy to find that Met37/Met39/Pro43/Met44 on the extracellular loops of DII/S1-S2 were also an important part of the combination of Syb-prII-1 and VSD2^rNav1.8^; this was in agreement with our previous findings [28]. Our analysis revealed the binding mode, important interaction regions, critical residues, and the specific interaction types between Syb-prII-1 and VSD2^rNav1.8^.

## 3. Discussion

So far, there have been many different views on the pathogenesis of TN. There are two widely accepted mechanism hypotheses: central pathogenesis and peripheral pathogenesis. The demyelination theory is the mainstream of the peripheral disease theory. TN can be divided into two categories according to the pathological mechanism. (1) TN animal model based on central pathophysiology. The trigeminal nerve root implantation model established by Burchiel et al. [29] could induce chronic inflammatory injury of TG. However, the damage is huge, and the behavioral method cannot be used to detect pain changes. Sakai et al. [30,31] established the model by injecting penicillin G-K, strychine, pictortoxin, and other central stimulant drugs into the subarachnoid space of guinea pigs, which was very similar to the clinical symptoms of TN. However, only acute symptoms of TN can be simulated; chronic symptoms and spontaneous pain characteristics of TN cannot be simulated. (2) TN animal model based on the theory of peripheral disease. Carragenan, formalin, and complete Freund’s adjuvant were injected subcutaneously into the face of rats to induce the spontaneous electrical activity increase in sensory neurons [32,33], which is suitable for TN studies caused by inflammation. The IoN-CCI model is a common model for studying TN and is also the model adopted by our laboratory. The infraorbital nerve branch of TG was exposed through the oral cavity, paranasal and upper eyebrow arch, respectively, and then, the infraorbital nerve was ligated with chrome gut. The Ion-CCI model is a chronic pain model, which can be used in the study of neuropathic pain and inflammatory pain.

The MAPKs pathway is closely related to neuronal plasticity in allodynia and pathological pain [34,35]. Lim et al. demonstrated that in the IoN-CCI model, the MAPKs pathway is associated with neuropathic pain [36]. In addition, in downstream molecules targeted by ERK5, the cAMP response element binding protein (CREB) also plays an important role in pain signaling [37]. The CREB-dependent gene expression is considered to be the key to central sensitization associated with persistent pain states. A dysfunction of MAPKs signaling pathway can be observed after tissue injury [38]. In neuronal cells, MAPKs can directly phosphorylate cellular proteins, activate downstream regulatory pathways, and finally increase the transcription process of pain-related genes [39]. After the establishment of the IoN-CCI model, partial degeneration and necrosis of trigeminal nerve and demyelination lesions were induced. We found that the phosphorylation level of MAPKs pathway protein changed to different degrees after modeling. The ERK5-targeted CREB signaling pathway plays a crucial role in pain [40,41]. After modeling, the phosphorylation levels of ERK5 and CREB proteins were significantly increased. Syb-prII-1 could significantly inhibit the elevation of P-CREB and relieve pain. Syb-prII-1 also inhibited the phosphorylation of ERK1/2, JNK, and p38 proteins in trigeminal nerve to varying degrees. Based on the effect of Syb-prII-1 on the phosphorylation level of MAPKs pathway, we inferred that Syb-prII-1 plays an important role in the regulation of TN by inhibiting the MAPKs signaling pathway.

Pathogenesis of neuropathic pain is regulated by multiple pathways. Voltage-gated sodium channels play a more direct role as the most downstream functional protein in neurons [42]. VGSCs were divided into tetrodotoxin-sensitive (TTX-S) sodium channels and tetrodotoxin-resistant (TTX-R) sodium channels. The former are blocked by nanomolar levels of tetrodotoxin, including Nav1.1, Nav1.2, Nav1.3, Nav1.4, Nav1.6, and Nav1.7. The latter, including Nav1.5, Nav1.8, and Nav1.9, require micromolar levels of tetrodotoxin to block them [20,21]. Nav1.7, Nav1.8, and Nav1.9 are expressed on peripheral neurons, such as TG and DRG, which are involved in the transmission of pain signals. Nav1.7 is mainly expressed on the nociceptors of small-diameter C fibers. Nav1.8 and Nav1.9 are mainly expressed in small-diameter C fiber and medium-diameter Aδ fiber neurons [43,44,45]. After investigation, we found that Nav1.8 and Nav1.9 were mainly distributed in small and medium-diameter neurons in TG, which was consistent with the reported results. Studies have shown that Nav1.8 and Nav1.9 have varying degrees of changes in neuropathic pain models [46,47]. Knocking out Nav1.8 or Nav1.9, the pain threshold of rats increased to varying degrees [48,49,50]. After the Ion-CCI model, we found that Nav1.8 significantly decreased, while Nav1.9 had a small change, and its trend was similar to that of Nav1.8, which was also consistent with the reports [16,17]. The reason for the decreased expression of Nav1.8 may relate to the damage of neuron cell body caused by peripheral nerve injury, which was consistent with the results of HE staining. Syb-prII-1 can further down-regulate Nav1.8 and Nav1.9 to reduce pain. Therefore, it can be inferred that the pathogenesis of neuropathic pain may closely relate to the down-regulation of Nav1.8, and Nav1.9 may play an auxiliary role in the pathogenesis of pain.

Nav1.8 and Nav1.9 subtypes are mainly distributed on peripheral small-diameter C fiber receptors and medium-diameter Aδ fiber receptor neurons. In order to further investigate the influence of Nav1.8 and Nav1.9 subtypes on neuropathic pain and the role of Syb-prⅡ-1, Nav1.8 and Nav1.9 were functionally investigated. Small and medium-diameter TG neurons were used as the research object for whole-cell patch clamp experiments. After modeling, the current density of Nav1.8 and Nav1.9 decreased to different degrees, and Nav1.8 was the most significant, which was consistent with previous experiments. After Syb-prⅡ-1 was given, it was found that the current density was also decreased in varying degrees, proving that Syb-prⅡ-1 has varying degrees of inhibition on Nav1.8 and Nav1.9. However, the change of current density cannot explain the cause of hyperalgesia and the analgesic mechanism of Syb-prⅡ-1. Tiber et al. [51] reported that scorpion β toxin was able to affect the steady-state activation and inactivation process of voltage-gated sodium ion channels. Therefore, the dynamic processes of Nav1.8 and Nav1.9 were investigated. The results showed that the steady-state activation curve of Nav1.8 drifted to the right, and the steady-state deactivation curve drifted to the left. It was speculated that the abnormal discharge of neurons after nerve injury caused by the modeling, which affected the inactivation process of Nav1.8, accelerated its entry into the resurrection state to increase the firing frequency of neurons, leading to increased neuronal excitability and hyperalgesia. The dynamic process of Nav1.9 was like that of Nav1.8, but there was no significant difference, indicating that it was not closely related to neuropathic pain. We speculated that it may play an auxiliary role. After Syb-prⅡ-1 was given, it was found that the steady-state activation curve of Nav1.8 drifted to the left, and the steady-state inactivation curve drifted to the right. This showed that the firing frequency of neurons was reduced, and abnormal firing was inhibited, thus exerting an analgesic effect. Most current studies have shown that analgesic drugs acting on voltage-gated ion channels almost all exert an analgesic effect in this way [52]. After Syb-prⅡ-1 was given, it was found that the reversal potential of Nav1.8 had changed significantly. Therefore, it can be speculated that Syb-prⅡ-1 is specific to the function of Nav1.8. This was also consistent with the computational simulation results.

We clarified the binding mode, critical residues, functions of the combination, and the specific interaction types between Syb-prII-1 and VSD2^rNav1.8^ by using the computational simulation methods. Based on the results mentioned above, we predicted that S12/L14/W35/W37 on Syb-prII-1 were the key residues of the interactions between Syb-prII-1 and VSD2^rNav1.8^, especially W37. These residues probably guaranteed the strong binding between Syb-prII-1 and VSD2^rNav1.8^. Residues on the β-turn also represent another set of essential components that ensure the biological activity of the ligand. Those results were also in agreement with our previous MD simulation results, where the residue W38 of AGAP exerted a crucial influence on the formation of IS1 and IS2 [53].

There were nine amino acid residues of Syb-prII-1 located at the binding surface of VSD2^rNav1.8^-Syb-prII-1 (termed IS1 and IS2, respectively). Residues K10, S12, L14 at the loop between α-helix and β-sheet I in Syb-prII-1 were distributed in IS1. Residues Y31, Y33, W35, W37, L39 at the β-turn were located in IS2. Based on the above analysis, we speculated that these interaction regions and amino acid residues were the main factors affecting the function of Nav1.8. 

In the follow-up study, the scorpion toxin could be modified accordingly to improve the specificity of Nav1.8. The molecular dynamics simulation experiment was only used to simulate the interaction between Syb-prII-1 and VSD2^rNav1.8^ in a real physiological environment. We will verify the interaction between Syb-prII-1 and Nav1.8 channel protein through GST pull-down and surface plasmon resonance experiments in subsequent experiments. Finally, the Nav1.8 chimera will be constructed, and the electrophysiological study will be performed again.

In general, Syb-prII-1 can inhibit TN by the MAPKs pathway, and it can also directly affect the expression of Nav1.8 and Nav1.9, and inhibit Nav1.8 current and dynamic processes, thereby inhibiting neuronal excitability to achieve the purpose of regulating pain. Therefore, Syb-prII-1 can comprehensively influence the process of TN signal transmission, and the analgesic effect is quite ideal. Syb-prII-1 has high specificity, stability, and biological activity, and it has a wide range of biological activities. It is a kind of drug with practical value and application prospects. Syb-prII-1 has great application value as a potential drug to treat TN.

## 4. Materials and Methods

### 4.1. Animals

The Sprague Dawley female rats weighing about 150–180 g were used in the experiment. Rats were housed for 24 h in a standard laboratory environment and maintained at a constant temperature over a 12:12 h light and dark cycle. All experimental operations were performed in accordance with the regulations of the Animal Ethics Committee of Shenyang Pharmaceutical University, China (SCXK (Liao) 2015-0001).

### 4.2. IoN-CCI Model

The rats were anesthetized with 1% sodium pentobarbital (6 mL/kg) for surgical procedures. A longitudinal 1 cm incision was made along the orientation of the mouth and nose at the level of the first molar on the right side of the mouth. The blunt dissection muscle exposed the trigeminal nerve facial branch and was ligated with 4-0 absorbable sutures spaced 2 mm apart. The incision was sutured, and penicillin was injected intraperitoneally to prevent postoperative infection in rats. The sham group was operated as above but without ligation. 

### 4.3. Behavioral Testing

#### 4.3.1. Mechanical Allodynia

The mechanical allodynia threshold of the rat face was measured using a Von Frey electronic pain meter. Three days before the operation, adaptive training was conducted on the rats. The rats with calm response to the training stimulus and intact facial hair and skin were selected for detection after the operation. The mechanical allodynia threshold recorded the day before the surgery was used as the baseline level for the test, which was then measured 3, 5, 7, 9, 11, 13, 14 days after surgery, and 0.5, 2, and 4 h after administration. 

#### 4.3.2. Thermal Withdrawal Latency

A thermal pain tester was used to measure the thermal withdrawal threshold of rats. After the rats’ beard pad was exposed to a high-intensity radiation beam, a rapid dodge reaction occurred. The time from the start of irradiation to the occurrence of the dodge response was recorded, which was the thermal withdrawal latency. In the experiment, the temperature of the thermal radiation beam was 50 °C, and the lamp source was 8 cm away from the test area. The thermal withdrawal latency recorded on the day before the operation was used as the basic level of the test, which was then detected 3, 5, 7, 9, 11, 13, 14 days after the operation, and 0.5, 2, 4 h after the administration. 

#### 4.3.3. Autonomous Behaviors of Pain

The rats were placed in a plexiglass observation cage, and the behavior of rats was recorded. Among them, a video of 5 min duration was randomly selected to observe the facial grooming frequency of the rats. Each occurrence of facial grooming was recorded as 1 point. 

#### 4.3.4. Fatigue Rotating Rod Experiment

Five days before surgery, fatigue rotating rod training allowed the rats to reach an adapted rotational speed of 40 rpm. The time each rat persisted for (up to 300 s) and the rotation speed at the time of the drop were recorded. Tests were performed before surgery, one day before dosing, two hours after dosing, and one day after dosing.

### 4.4. Gait Analysis

Gait analysis training was performed 7 days before surgery. The trained rats were able to adapt to the gait analyzer speed (24 cm/min). Before the operation, the running state of the rats on the gait analyzer at a speed of 24 cm/min was recorded as a blank horizontal baseline. After surgery, the gait video of the rats was recorded again one day after the administration, two hours after the administration, one day after the administration. Test indicators were shown in Table 2.

### 4.5. HE Staining of Trigeminal Nerve

Rats were selected on the 7th, 15th, and 22nd day after modeling and were sacrificed after deep anesthesia. Complete trigeminal nerve tissue was taken out. The tissues were placed in 4% paraformaldehyde for 24 h and then transferred to 0.4% formalin solution or 75% ethanol solution for preservation, being stored at 4 °C. The tissues were dehydrated, transparent, and embedded to make paraffin sections. Paraffin sections of the trigeminal nerve tissue were baked in an oven at 60 °C for 2–4 h, after which the sections were deparaffinized and stained. The nucleus was stained blue with hematoxylin. The cytoplasm was stained with eosin to a different degree of red, and the calcium salt could be dyed blue or blue-violet. 

### 4.6. Western Blot

Total protein of trigeminal nerve was extracted, and the protein concentration was determined with the BCA method. The soluble antigen (target protein) was separated with 10% denatured polyacrylamide gel electrophoresis (SDS-PAGE). After separation, the protein was transferred from the gel to the PVDF membrane. It was blocked with 5% skim-milk-powder-TBST buffer for 1 h at room temperature; then, the residual buffer was washed with TBST. It was incubated in the primary antibody overnight at 4 °C. The next day, after washing with TBST buffer, a secondary antibody was added. Then, the secondary antibody was incubated for 1 h at room temperature, and the membrane was washed. The target protein was detected by chemical imaging with the ECL kit. 

### 4.7. Immunofluorescence of Trigeminal Nerve Tissue

The paraffin sections were placed in order of xylene I, xylene II, absolute ethanol I, absolute ethanol II, 95% ethanol, 85% Ethanol, 75% ethanol, 50% ethanol. They were washed with PBS; 10% goat serum (20 μL) was added dropwise to each tissue and sealed in a wet box at 37 °C for 1 h in the dark. The serum was removed, and a mixture of rabbit anti-Nav1.8/Nav1.9 antibody (1:200) and mouse anti-NF200 (NF200 is a subunit of neurofilament and can be used as a marker for large-diameter neurons) antibody (1:200) were added and stored in a refrigerator at 4 °C overnight. The next day, they were washed with PBST. An amount of 20 μL of secondary antibody mixture was added dropwise in the dark, incubated for 1 h at room temperature, and washed with PBST. Finally, a fluorescent anti-quenching agent was added for observation.

### 4.8. Patch Clamp

The trigeminal ganglia of the rats were placed in an ice bath DMEM/F12 medium and transferred to the clean bench. Microscopical forceps were used to remove the nerve surface fascia, and the TG was cut up and transferred to a flask containing 1.5 mL of digestive fluid with ophthalmic scissors. Quickly, it was put into an incubator at 37 °C for digestion for 30 min and gently shaken every 3 min to ensure full contact between the tissues and digestive juice. The digestive juice was then absorbed and washed with serum-containing culture solution 3 times to terminate the digestive reaction. It was then transferred to a centrifuge tube, gently blown 15 times to disperse the tissue, allowed to stand for 2 min, and 0.3 mL culture solution was added. It was blown with a 200 μL pipette gun 3–5 times and allowed to stand for 1 min. Air bubbles should not be produced during blowing. After tissue deposition, the TG neuron cell suspension was sucked out and planted in a culture dish coated with 0.01% polylysine. An amount of 0.3 mL of cell culture solution was added, and the above steps were repeated until the tissue disappeared. Petri dishes were placed in an incubator containing 5% CO_2_/95% O_2_ at 37 °C for 1–2 h for patch clamp experiment.

Whole-cell patch clamp technique was performed at 22–25 °C. The patch clamp electrode was pulled with P-97, and the resistance was 3–5 MΩ. Extracellular fluid (mM) recording Nav1.8/Nav1.9 sodium current: choline chloride 100, NaCl 40, KCl 3, CaCl_2_ 2.5, MgCl_2_ 1, HEPES 10, glucose 10, LaCl_3_ 0.1, TTX 1000 nM. Then, the pH was adjusted to 7.3 with 1 mol/L NaOH and stored at 4 °C. Electrode solution (mM) for recording Nav1.8/Nav1.9 sodium current: CsCl 100, CsF 30, NaCl 8, CaCl_2_ 2.4, MgCl_2_ 1, EGTA 5, HEPES 10, Na_2_ATP 4, GTP 0.4. Then, the pH was adjusted to 7.3 with 1 mol/L CsOH, filtration, and packing and stored at −20 °C. The patch clamp was an Axon Patch 200B amplifier (Axon Instruments, San Jose, 55101, USA); the signal was filtered at 1 KHz; and the acquired data were digitally stored on a computer for further analysis. The current was recorded as follows.

Nav1.8 current: After cell membrane breaking, it was stable for 2–3 min. The 1000 nM TTX was used to block Tetrodotoxin-sensitive (TTX-S) sodium channels. The cell was held at −100 mV and then depolarized to −50 mV for 500 ms to shield the Nav1.9 current. Then, a depolarized stimulated square wave with a wave width of 100 ms and a step of 10 mV was given, increasing from −50 mV to +50 mV, and finally returning to the clamping voltage of −100 mV, thus recording the Nav1.8 current. Nav1.9 current: Cells were held at −100 mV. Then, a depolarized stimulated square wave with a wave width of 100 ms and a step of 5 mV, increasing from −80 mV to −35 mV, was given. Finally, they were returned to the holding voltage of −100 mV. This could be recorded with the Nav1.9 current.

Nav1.8 deactivation current: Cells were held at −100 mV, giving a depolarized square wave with a wave width of 500 ms and a step of 10 mV, increasing from −120 mV to 0 mV. Then, a test voltage of 0 mV for 100 ms was applied. Finally, cells were returned to a clamping voltage of −100 mV, and the Nav1.8 deactivation current was recorded. Nav1.9 deactivation current: Cells were held at −100 mV, then given a depolarized square wave with a wave width of 700 ms and a step size of 5 mV, increasing from −110 mV to −35 mV. Then, a test voltage of −50 mV for 100 ms was applied, and finally, cells were returned to a clamping voltage of −100 m. The Nav1.9 deactivation current was recorded.

### 4.9. Homology Modeling and Molecular Docking

Sequence alignment was carried out for the best template to model a 3D structure of VSD2^rNav1.8^ and Syb-prII-1 using ClustalX 2.1 [54]. In total, 500 conformations were built with Modeller 9.9 [55], and then, the models were ranked based on the DOPE score. Further validations were performed with ERRAT [56,57], PROCHECK [38], and WHATCHECK [58,59]. 

Molecular docking was performed with the ZDOCK module [60]. The results were analyzed in a report to obtain the possible combination mode of Syb-prII-1 and VSD2rNav1.8. The angular step was set at 6 Å to avoid missing the best binding poses and obtain sufficient binding configurations. Finally, 2000 poses were generated. Based on the RMSD cut-off, 2000 poses were divided into 60 clusters. Poses belonging to the largest cluster and with the strongest binding free energy were picked as the initial configurations for MD simulation. After screening the results of ZDOCK, RDOCK optimization was performed to calculate the corresponding energy value [61]. 

### 4.10. Molecular Dynamics Simulation

GROMACS 2018 package was used to simulate the interaction between Syb-prII-1 and VSD2rNav1.8 in a real physiological environment [62]. Periodic boundary conditions were applied to ensure the rationality of the atomic force [63]. Topology files of all the systems were generated with the GROMOS-53a6 force field [64]. VSD2^rNav1.8^ is a transmembrane protein; thus, the 1-palmitoyl-2-oleoyl-sn-glycero-3-phosphocholine (POPC) bilayer model [65] was used. After adjusting the center of POPC and the proteins in the appropriate coordinates, the inflated GRO methodology [65] was applied to pack the lipids around the protein. VSD2^rNav1.8^ was accurately embedded within the phospholipid bilayer. The water box produced by the SPC water model [66] was introduced to solvate the systems, and counterions were added to maintain their electroneutrality. The system was heated from 0 K to 310 K using the modified Berendsen thermostat [67] with position restraints on the complex after minimization. Given that the rise in temperature would increase the thermodynamic motion of the systems, NPT was carried out for 1 ns to let the systems equilibrate after heating steps. Subsequently, MD simulation of 100 ns was performed at 310 K, 1 atm, and a time step of 2 fs. Long-range electrostatic interactions were assessed with the particle mesh Ewald method [68], whereas linear constraint solver (LINCS) was used to reset all bonds to the correct lengths [69]. Tools from the GROMACS package, PyMOL [70], and VMD [71] were used to analyze the trajectories and constructs obtained through MD.

### 4.11. Materials and Regents

Syb-prII-1 was expressed by Professor Zhang Jinghai of Shenyang Pharmaceutical University using DNA recombination technology and PCR amplification technology, and its purity was over 95%. It should be diluted with 0.9% physiological saline before use. Electronicvon frey anesthesiometer and plantar test (Hargreaves Method) & tail flick meter in one unit were purchased from IITC Inc. Life Science (New York, NY, USA); Paraffin slicer was purchased from Leica (Shanghai, China); Patch clamp amplifier 200 B and D-A conversion system 1440a were purchased from Axon Instruments (San Jose, CA, USA); Electrode drawing machine was purchased from Sutter Instrument (San Francisco, CA, USA); 3D hydraulic micromanipulator was purchased from NARISHIGE (Tokyo, Japan); Fatigue rotary rod meter was purchased from Ugo Basile biological research apparatus company (Milan, Italy); Gait analysis system was purchased from MiniSun LLC (Hefei, China); Low temperature high speed centrifuge and CO_2_ constant-temperature incubator were purchased from Thermo Fisher Scientific (Shanghai, China); SDS-PAGE system were purchased from Bio-Rad Laboratories, Inc. (Shanghai, China); Penicillin sodium for injection and morphine hydrochloride injection were purchased from Shenyang First Pharmaceutical Co. LTD (Shenyang, China); Medical chromium gut thread was purchased from Shanghai Pudong Jinhuan Medical Supplies Co., LTD (Shanghai, China); Xylene and analysis of alcohol were purchased from Shenyang Chemical Reagent Factory (Shenyang, China); HE staining kit, horse serum and BSA bovine serum protein were purchased from Solarbio (Beijing, China); Protein lysates were purchased from Thermo Fisher Scientific (Shanghai, China); PMSF, acrylamide, methyl acrylamide, TEMED, APS, SDS, glycerin, tween-20, bromophenol blue and tris-base were purchased from Amresco Inc. (Solon, OH, USA); Streptomycin sulfate for injection was purchased from Shandong Lukang Pharmaceutical Co., LTD (Jining, China); DMEM/F12 medium and fetal bovine serum were purchased from Gibco|Thermo Fisher Scientific (Shanghai, China); Glutamine was purchased from Biosharp (Hefei, China); Tetrodotoxin was purchased from Hebei Fishery science and technology development company (Hebei, China); NaCl, CaCl_2_, MgCl_2_ and NaOH were purchased from Tianjin Bodi Chemical Co., LTD (Tianjin, China); KCl and KH_2_PO_4_ were purchased from Xilong chemical factory (Shantou, China); Na_2_HPO_4_·12H_2_O was purchased from Tianjin Damao chemical reagent factory (Tianjin, Beijing). NF 200 antibodies, L-polylysine, HEPES, LaCl_3_, CsCl, CsF, EGTA, GTP, Na_2_ATP trypsin, collagenase type IA, DNase enzyme type IV, choline chloride and CsOH were purchased from Sigma Inc (St. Louis, MO, USA); BCA protein concentration Kit, ECL chemiluminescence Kit, GAPDH, Cy_3_ labeled sheep anti-rabbit IgG, FITC labeled sheep anti-mouse IgG and anti-fluorescence quenching agent were purchased from Biyuntian Biotechnology Co., LTD (Shanghai, China); PageRuler Plus prestained protein was purchased from Fermentas Inc. (Shanghai, China); Lane marker sample buffers were purchased from Thermo Fisher Scientific (Shanghai, China); Horseradish enzyme labeled goat anti-mouse IgG, horseradish enzyme labeled goat anti-rabbit IgG, PBS phosphate buffer and citrate solution were purchased from Zhongshan Golden Bridge Co., LTD (Beijing, China); Sodium orthovanadate was purchased from Beijing Chemical Reagent Inc.(Beijing, China); NaF was purchased from Beijing Bodi Chemical Co., LTD (Tianjin, China); Skim milk powder was purchased from BD Inc. (Shanghai, China); Anti-Nav1.8 antibodies and anti-Nav1.9 antibodies were purchased from Alomone Inc. (Shanghai, China); Anti-p-ERK5, anti-ERK5, anti-p-CREB and anti-CREB were purchased from Cell Signaling Inc. (Shanghai, China).

### 4.12. Statistical Analysis

ClustalX 2.1 was used for sequence alignment. Conformations were built with Modeller 9.9. GROMACS 2018 package was used to simulate the interaction between Syb-prII-1 and VSD2rNav1.8 in a real physiological environment. The pCLAMP 10.0 software (Axon Instruments, San Jose, CA, USA) was used to acquire and analyze the data. SPSS 22.0, ImageJ, Origin 8.0, and Graphpad Prism 5.01 software were used for statistical analysis and plotting the data. One-way ANOVA and repeated one-way ANOVA were used for comparison between groups. All data were expressed as mean ± SEM. *p* < 0.05 was considered significant.

## Figures and Tables

**Table 1:** Residues involved in the binding surface of VSD2^rNav1.8^-Syb-prII-1 and the types of interaction bonds.

Regions on Syb-prII-1	Residues on Syb-prII-1	Residues on VSD2^rNav1.8^	Types of Interaction Bonds
loop between	K10	Gly105	Hydrogen bond
α-helix and β-sheet I	S12	Lys104	Hydrogen bond
		Leu107	Hydrogen bond
	L14	Leu97	Hydrophobic
		Ala101	Hydrophobic
		Leu107	Hydrophobic
α-helix	N17	Met44	Hydrogen bond
β-turn	Y31	Pro43	Pi-Alkyl
	Y33	Met44	Pi-Sulfur
	W35	Met39	Hydrogen bond
		Met37	Hydrogen bond
	W37	Leu110	Hydrogen bond
		Glu98	Hydrogen bond
		Arg114	Pi-Cation
		Arg114	Pi-Cation
	L39	Val109	Alkyl

Residues on Syb-prII-1 are marked with a character; Residues on VSD2^rNav1.8^ are marked with three characters.

**Table 2:** Indices of digital gait.

Parameter	Unit	Definition
Stride length	cm	A paw spans the length of a specific stride
Paw area	cm^2^	Paw area recorded at peak ground contact
Stance duration	s	Divided into breaking duration and propulsion duration. Breaking duration describes the time required for the animal’s limbs to decelerate and control the paw to touch the ground; Propulsion duration describes the time required for the animal’s limbs to accelerate and accelerate the starting process.

## Data Availability

Not applicable.
